# Supplementary material for: Serum hepatitis B core antibody as the prognostic factor for diffuse large B-cell lymphoma
Source: Microbiol Spectr. 2025 Mar 25;13(5):e03170-24. doi: 10.1128/spectrum.03170-24 (PMC12053999; doi:10.1128/spectrum.03170-24)
Supplement: Supplemental figures — Fig. S1 to S4. [file spectrum.03170-24-s0001.docx]

**Supplementary Figure S1-4**


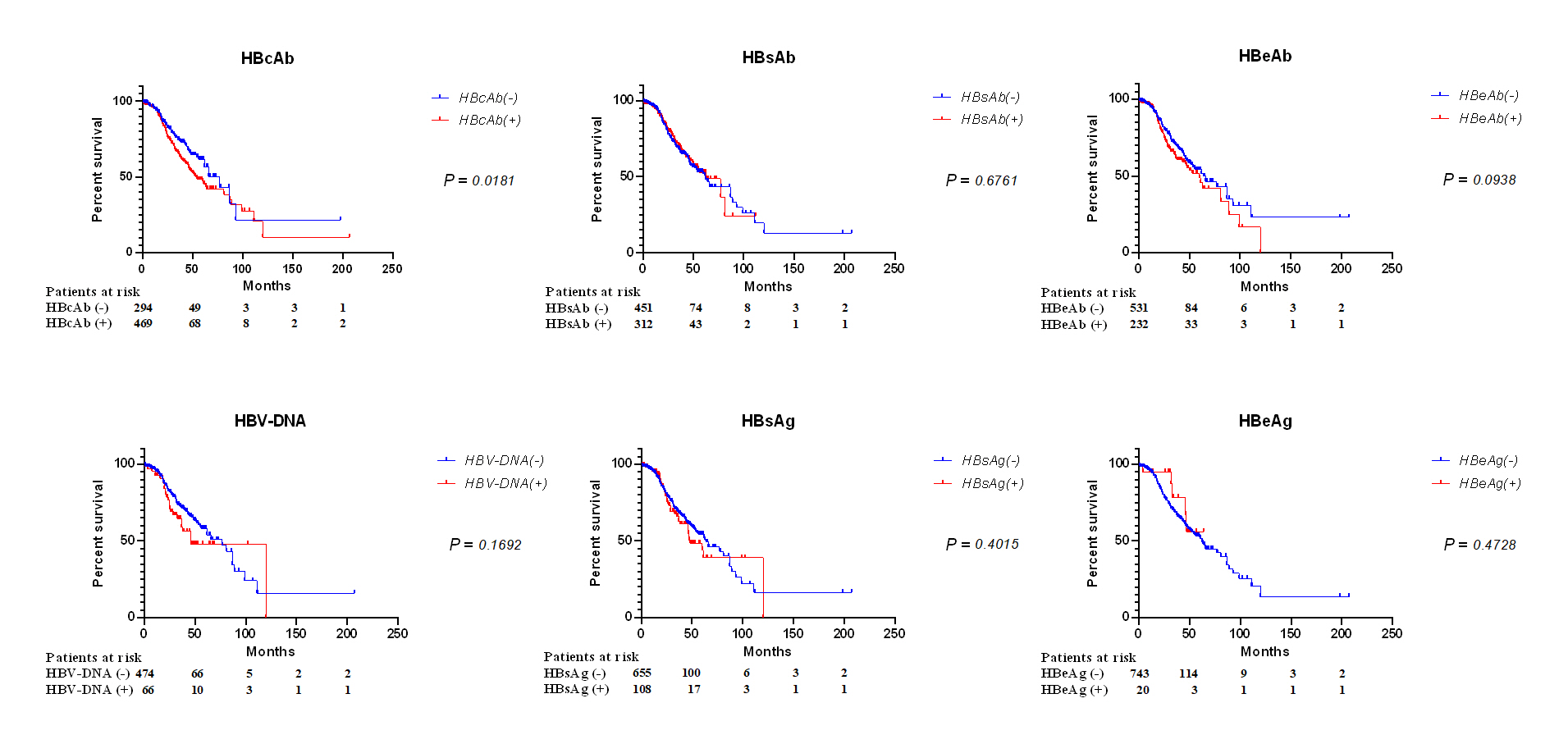
Figure S1. OS of DLBCL patients with different serum HBV status.

A total of 1,062 DLBCL patients followed up were grouped according to their serum HBV status: HBcAb, HBsAb, HBeAb, HBV-DNA, HBsAg, and HBeAg. The difference in OS rates among these groups was compared through survival analysis.


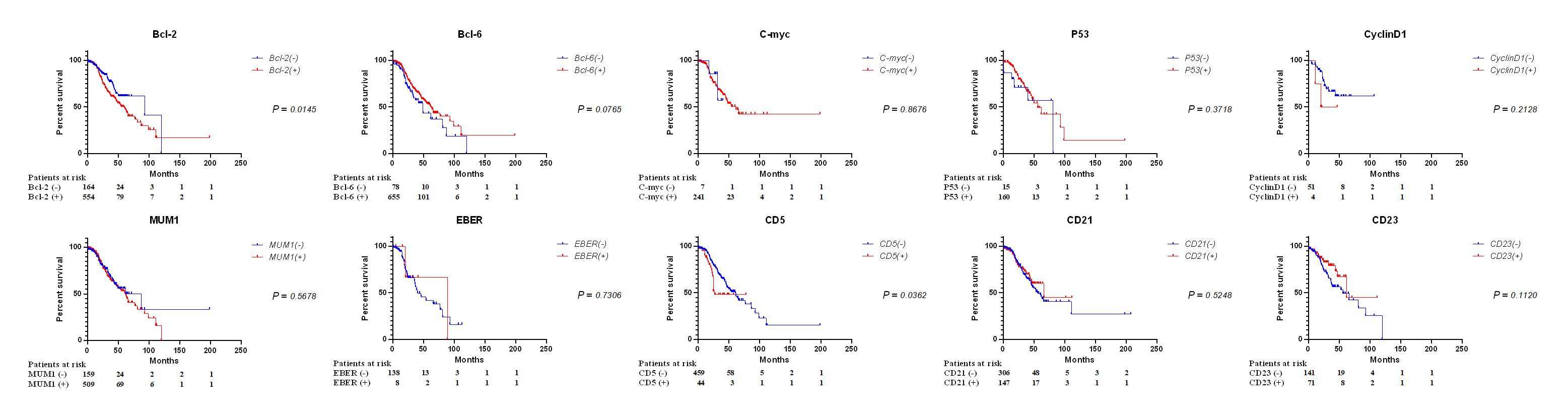
Figure S2. OS of DLBCL patients with different protein status in DLBCL tissues.

The IHC results of follow-up DLBCL patients were analyzed, and the patients were grouped according to the qualitative status of DLBCL tissue proteins, including Bcl-2, Bcl-6, C-myc, P53, CyclinD1, MUM1, EBER, CD5, CD21, CD23 etc., and the differences in OS rates between groups were compared through survival analysis.


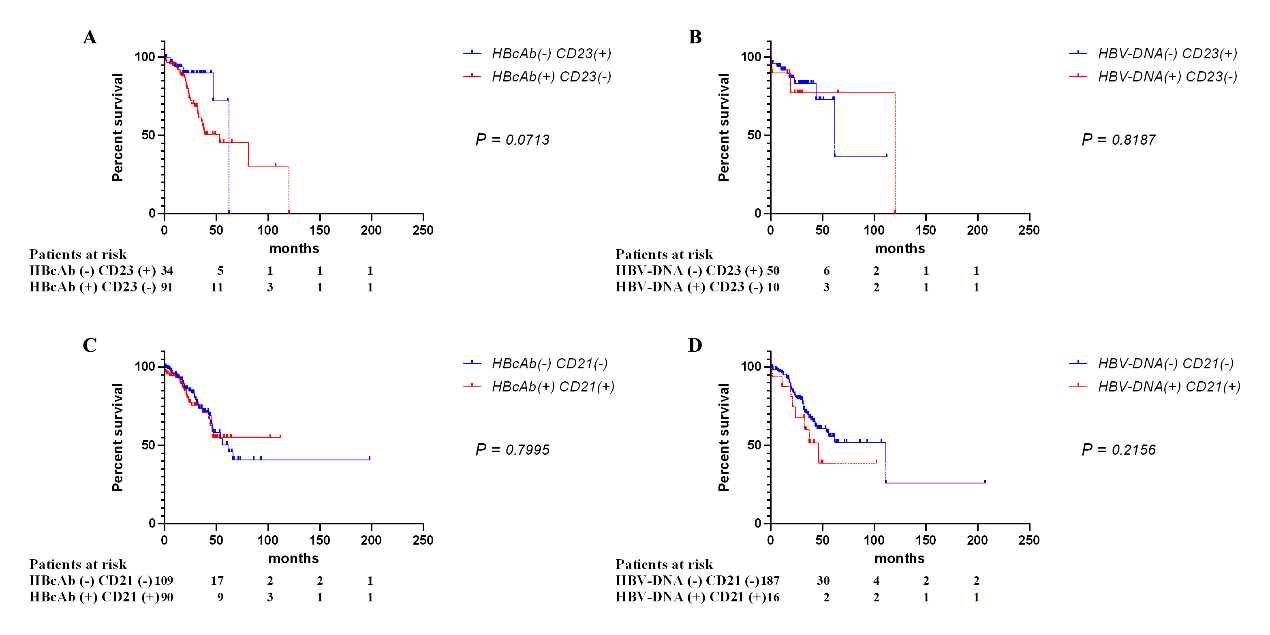


Figure S3. OS of DLBCL patients combined with HBV indicators and tissue protein expression.

Follow-up patients with DLBCL were grouped according to HBcAb (A) and HBV-DNA (B) combined with tissue CD23 expression, and HBcAb (C) and HBV-DNA (D) combined with tissue CD21 expression. The OS rates between groups were compared by survival analysis.


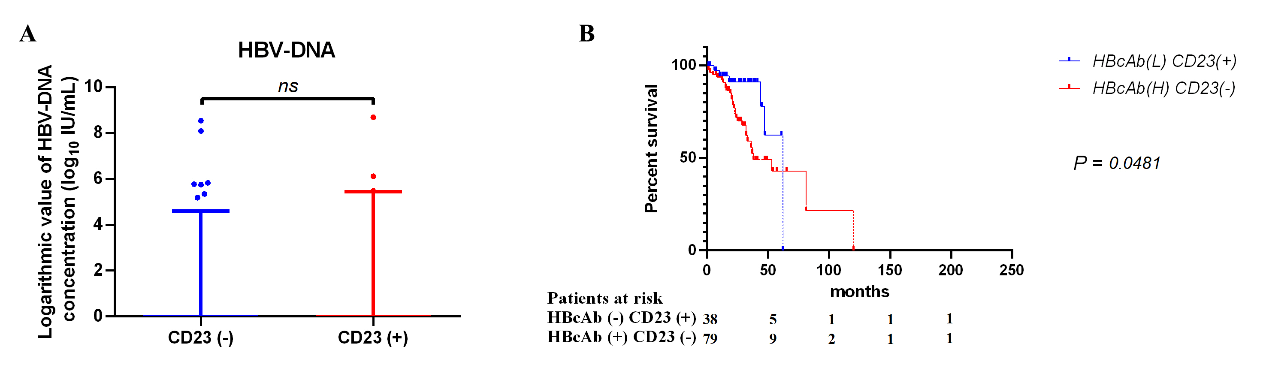


Figure S4. Correlation between HBV-DNA concentration and tissue CD23 expression, and OS of DLBCL patients in combination with HBcAb concentration and CD23 status.

The correlation between HBV-DNA concentration and CD23 in DLBCL patients was compared by the Mann-Whitney method, which was illustrated by the box of median with quartile and whiskers of 2.5-97.5 percentile (A). Patients were classified according to the median concentration of HBcAb and CD23 status, and the OS rates among groups were compared through survival analysis (B).
