# Supplementary material for: Serum hepatitis B core antibody as the prognostic factor for diffuse large B-cell lymphoma
Source: Microbiol Spectr. 2025 Mar 25;13(5):e03170-24. doi: 10.1128/spectrum.03170-24 (PMC12053999; doi:10.1128/spectrum.03170-24)
Supplement: Table S1 — Correlation between tissue protein expression and all HBV indicators detected in DLBCL. [file spectrum.03170-24-s0002.docx]

| **Supplementary Table S1**  Table S1. Correlation between tissue protein expression and all HBV indictors detected in DLBCL. | | | | | | | | | | | | | | | | | | | | | | | | | | |
| --- | --- | --- | --- | --- | --- | --- | --- | --- | --- | --- | --- | --- | --- | --- | --- | --- | --- | --- | --- | --- | --- | --- | --- | --- | --- | --- |
| **IHC** | | **HBV** | | | | | | | | | | | | | | | | | | | | | | | |  |
|  |  | HBcAb | | |  | HBeAb | | |  | HBsAb | | |  | HBV-DNA | | |  | HBsAg | | |  | HBeAg | | |  |  |
|  |  | (-) | (+) | *P* |  | (-) | (+) | *P* |  | (-) | (+) | *P* |  | (-) | (+) | *P* |  | (-) | (+) | *P* |  | (-) | (+) | *P* |  |  |
| Bcl-2 | (-) | 272 | 376 | 0.105 |  | 466 | 182 | 0.322 |  | 378 | 270 | 0.474 |  | 315 | 38 | 0.169 |  | 568 | 80 | 0.063 |  | 632 | 16 | 0.581 |  |  |
|  | (+) | 801 | 1284 |  |  | 1457 | 628 |  |  | 1183 | 902 |  |  | 948 | 149 |  |  | 1766 | 319 |  |  | 2025 | 60 |  |  |  |
| Bcl-6 | (-) | 133 | 197 | 0.589 |  | 236 | 94 | 0.635 |  | 193 | 137 | 0.454 |  | 147 | 23 | 0.723 |  | 282 | 48 | 0.998 |  | 320 | 10 | 0.809 |  |  |
|  | (+) | 943 | 1490 |  |  | 1709 | 724 |  |  | 1370 | 1063 |  |  | 1141 | 164 |  |  | 2079 | 354 |  |  | 2365 | 68 |  |  |  |
| C-myc | (-) | 21 | 22 | 0.203 |  | 33 | 10 | 0.309 |  | 25 | 18 | 0.804 |  | 26 | 3 | 1.000^a^ |  | 40 | 3 | 0.147 |  | 42 | 1 | 1.000^a^ |  |  |
|  | (+) | 387 | 602 |  |  | 687 | 302 |  |  | 556 | 433 |  |  | 430 | 56 |  |  | 841 | 148 |  |  | 958 | 31 |  |  |  |
| P53 | (-) | 17 | 36 | 0.249 |  | 38 | 15 | 0.670 |  | 26 | 27 | 0.216 |  | 26 | 2 | 0.558^a^ |  | 44 | 9 | 0.724 |  | 53 | 0 | 0.387^a^ |  |  |
|  | (+) | 254 | 379 |  |  | 436 | 197 |  |  | 366 | 267 |  |  | 284 | 45 |  |  | 537 | 96 |  |  | 615 | 18 |  |  |  |
| CyclinD1 | (-) | 95 | 185 | 0.416^a^ |  | 188 | 92 | 1.000^a^ |  | 150 | 130 | 1.000^a^ |  | 104 | 16 | 1.000^a^ |  | 237 | 43 | 0.240^a^ |  | 272 | 8 | 1.000^a^ |  |  |
|  | (+) | 3 | 3 |  |  | 4 | 2 |  |  | 3 | 3 |  |  | 6 | 0 |  |  | 4 | 2 |  |  | 6 | 0 |  |  |  |
| MUM1 | (-) | 255 | 357 | 0.122 |  | 432 | 180 | 0.952 |  | 359 | 253 | 0.216 |  | 300 | 54 | 0.088 |  | 517 | 95 | 0.322 |  | 594 | 18 | 0.805 |  |  |
|  | (+) | 735 | 1191 |  |  | 1362 | 564 |  |  | 1075 | 851 |  |  | 858 | 114 |  |  | 1658 | 268 |  |  | 1873 | 53 |  |  |  |
| EBER | (-) | 410 | 605 | 0.381 |  | 694 | 321 | 0.302 |  | 543 | 472 | 0.719 |  | 335 | 37 | 0.723^a^ |  | 880 | 135 | 0.916 |  | 994 | 21 | 0.656^a^ |  |  |
|  | (+) | 32 | 38 |  |  | 52 | 18 |  |  | 39 | 31 |  |  | 21 | 3 |  |  | 61 | 9 |  |  | 68 | 2 |  |  |  |
| CD5 | (-) | 724 | 1143 | 0.346 |  | 1316 | 551 | 0.332 |  | 1034 | 833 | 0.750 |  | 859 | 132 | 0.640 |  | 1592 | 275 | 0.824 |  | 1809 | 58 | 0.651 |  |  |
|  | (+) | 70 | 128 |  |  | 133 | 65 |  |  | 112 | 86 |  |  | 79 | 14 |  |  | 170 | 28 |  |  | 193 | 5 |  |  |  |
| CD21 | (-) | 463 | 753 | 0.900 |  | 839 | 377 | 0.642 |  | 677 | 539 | 0.591 |  | 571 | 76 | 0.187 |  | 1035 | 181 | 0.408 |  | 1177 | 39 | 0.424 |  |  |
|  | (+) | 238 | 392 |  |  | 428 | 202 |  |  | 359 | 271 |  |  | 309 | 53 |  |  | 527 | 103 |  |  | 614 | 16 |  |  |  |
| CD23 | (-) | 241 | 383 | **0.003** |  | 432 | 192 | 0.052 |  | 340 | 284 | 0.272 |  | 294 | 43 | 0.998 |  | 530 | 94 | 0.724 |  | 603 | 21 | 0.548 |  |  |
|  | (+) | 118 | 119 |  |  | 180 | 57 |  |  | 139 | 98 |  |  | 123 | 18 |  |  | 199 | 38 |  |  | 227 | 10 |  |  |  |
| The association between HBV indicators and various DLBCL tissue molecules in DLBCL patients was compared using the Chi-square test. The significance level of *P*<0.05 was indicated in bold.  ^a^ When the expected frequency was less than 5, the exact probability method was applied. Due to incomplete histochemistry results for some diagnosed DLBCL patients, the total number of individual histochemistry results was actually ≤4491 cases. | | | | | | | | | | | | | | | | | | | | | | | | | | |
